# Farm Crops Depredation by European Bison (*Bison bonasus*) in the Vicinity of Forest Habitats in Northeastern Poland

**DOI:** 10.1007/s00267-012-9913-7

**Published:** 2012-07-28

**Authors:** Emilia Hofman-Kamińska, Rafał Kowalczyk

**Affiliations:** Mammal Research Institute, Polish Academy of Sciences, Gen. Waszkiewicza 1c, 17-230 Białowieża, Poland

**Keywords:** Białowieża Primeval Forest, Conservation management, Crop selection, Damage compensation, Seasonal migrations, Wildlife-human conflict

## Abstract

European bison, the largest mammal in Europe, after being exterminated in the wild and then restored during the 20th century is still listed by the International Union for Conservation of Nature (IUCN Red List of Threatened Species) as a species vulnerable to extinction. However, the increasing number of European bison, through creation of new and expansion of existing populations strongly increases the risk of human-bison conflict in the near future. We analyzed the depredation of farm crops by bison and the factors influencing the level of damage in the vicinity of two forest areas inhabited by bison in northeastern Poland. Between 2000 and 2010, the total cost of compensation was € 196,200. The level of damage and amount of compensation was increasing from year to year in both forests and correlated with the number of bison. The majority of damage (57 % of cases) was recorded in winter (December–March). Snow depth and temperature did not influence the frequency of damage. The incidences of damage increased with decreasing distance from the woodland patches, therefore, 69 % of cases in Białowieża Forest, and 80 % in Knyszyn Forest were recorded closer than 0.5 km from nearest woodland patch. The majority of the crops damaged by bison were cereals (61 %) but also hay (20 %) and rape (13 %). When compared to the availability of crops, bison strongly selected rape and rye in both regions. This study is the first addressing the increasing problem of human-bison conflict in re-introduced populations and analyzing long-term data on crop depredation. Such situations probably occur in the majority of growing and expanding bison populations, however, it has not yet to be monitored and is rather neglected in post-Soviet countries.

## Introduction

Large wild herbivores have a major impact on their environment and in many parts of the world are the main sources of wildlife-human conflicts (Putman 1996; Putman and others 2011; Reimoser and Putman 2011; Smit and Putman 2011). They need extended areas and rich food resources (Distefano 2005; Osborn and Hill 2005), so the conflicts most often include competition for space and food with domestic animals (Prins 2000; Young and others 2005) and crop depredation (Herrero and others 2006; Osborn 2004; Putman and Moore 1998; Schley and others 2008; Thapa 2010) but also transmission of diseases (Ferroglio and others 2011; Kilpatrick and others 2009; Schmitt and others 2002), traffic collisions (Bissonette and others 2008; Bruinderink and Hazebroek 1996; Langbein and others 2011; Langley and Mathison 2008) and in most extreme cases loss of human life (Langbein and others 2011; Post 2000; Walpole and others 2003; Zhang and others 2006). Due to strong fragmentation and limited area of optimal habitats, distribution of large animals is in most cases limited to protected areas, often surrounded by farmlands and human settlements. This increases the risk of conflict with large herbivores, which often expand into those areas. In effect, areas adjacent to national parks and other protected areas are characterized by the highest levels of human-wildlife conflict (Linkie and others 2007; Naughton-Treves 1998; Plumb and others 2009; Rao and others 2002). The main efforts of wildlife managers in such areas are aimed at the reduction of these conflicts and the extension of protected areas (Hedges and Gunaryadi 2009; Nishi and others 2002; O’Connell-Rodwell and others 2000; Nyirenda and others 2011).

Large animals may cause huge economic losses in agriculture and forestry (Apollonio and others 2010; Liberg and others 2010; Maillard and others 2010; Ruusila and Kojola 2010). In Poland alone, the amount of compensation paid for crop damage by wild ungulates (mainly wild boar and red deer) in 2010 was 57.4 million PLN, (€ 13.7 million) (Central Statistical Office 2011). Increasing number of ungulates in Europe (Putman and others 2011), indicates there will be an increase in human-wildlife conflict and the costs of crop depredation.

The European bison (*Bison bonasus*), Europe’s largest mammal, historically inhabited the central and eastern part of the continent (Benecke 2005). Until the middle ages, the species survived only in isolated pockets due to habitat loss and culling (Pucek and others 2004). The last two bison populations existed in the 19th century in the Białowieża Forest and Caucasus mountains, were exterminated in 1919 and 1926 respectively (Krasińska and Krasiński 2007; Sztolcman 1926). The population was restored in captivity from zoo and breeding centre survivors and re-introduced into the wild in the second half of the 20th century to nearly 30 locations in Eastern Europe (Krasińska and Krasiński 2007; Pucek and others 2004). The current global European bison population is approx. 2,800 free-ranging individuals (1,030 in Poland), however, only seven populations comprise of more than 100 individuals (Krasińska and Krasiński 2010; Raczyński 2010). All bison populations were originally re-introduced into forest habitat, but over 70 % have expanded their range to include open (mainly agriculture) habitats (Kerley and others 2012). Supplementary feeding and culling are the main management practices, which are aimed at reducing the number of bison migrating out of forest habitats and damage to agriculture and the tree stands by bison.

Northeastern Poland, which is inhabited by three bison populations (650 individuals in total), constitutes the core of the global bison population. The amount of compensation paid to farmers for bison damage in this area is increasing from year to year and in 2010 cost nearly 362,000 PLN (over € 90,000) (data of Regional Directorate for Environmental Protection in Białystok). Despite the efficient compensation system, the presence of bison in this region is still not fully accepted by local communities.

Damage of crops by bison is recognized as quite a new aspect in bison management in Europe and as yet has not been analyzed. However, the increasing number of bison and the expected expansion of bison populations out of forest habitats, as well as the potential creation of new free-ranging herds, may increase the risk of human-bison conflict in the future (Hofman-Kamińska and Kowalczyk 2010). Additionally, Poland’s accession to EU and related agricultural subsidies caused intensification of farmland activities in previously abandoned or less intensively utilized fields and meadows adjacent to forests occupied by bison.

The aim of this paper was to analyze the amount, distribution and structure of crop damage caused by bison in the vicinity of the forest habitat they inhabit. We also asked what factors would influence the amount and distribution of damage in two bison populations differing in density, habitat conditions, management and farming practices. Finally, we discussed possible management actions to reduce amount of depredation to farm crops by this large herbivore.

## Materials and Methods

### Study Area

The study was conducted in northeastern Poland, in the Białowieża Forest and Knyszyn Forest inhabited by free-ranging populations of European bison. The other species of wild ungulates occurring in both areas are red deer (*Cervus elaphus*), roe deer (*Capreolus capreolus*), wild boar (*Sus scrofa*) and moose (*Alces alces*).

**Fig. 1:**
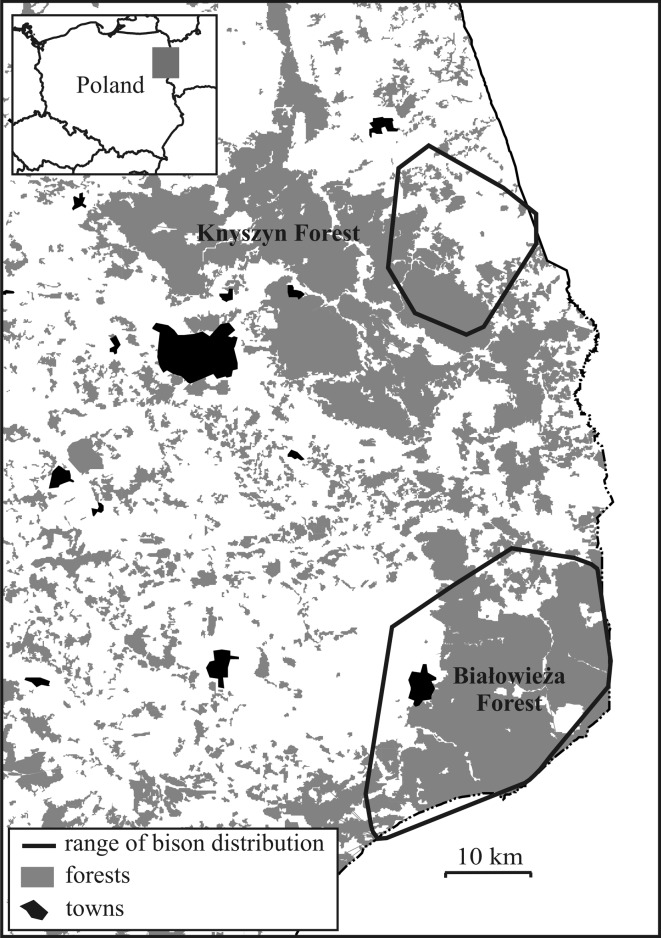
European bison distributions in Białowieża Forest and Knyszyn Forest in northeastern Poland

The Białowieża Forest [BF] (52°29′–52°N, 23°31′–24°21′E) is one of the few remaining preserved lowland forest in Europe, located on the Polish-Belarussian border (Fig. 1). The Polish part of the forest covers nearly 600 km^2^. Deciduous and mixed tree stands (mainly pine [*Pinus sylvestris*] (27 %), spruce [*Picea abies*] (25 %), alder [*Alnus glutinosa*] (20 %), and oak [*Quercus robur*] (12 %)) cover 94 % of the BF, while open habitats (glades with meadows, riversides, open sedge and reed marshes) constitute the remaining 6 % of BF (Sokołowski 2004). From the west and north, BF is surrounded by open habitats dominated by pastures and meadows (23.8 %) and arable lands (47.5 %), interrupted with small woodlands (25.1 %). The main cultivated crops are cereals [mainly rye (23 %) and oat (13 %)]. The proportion of arable lands covered by winter grains and rape is 41 %. In many areas hay is stored on meadows and often stays there for the whole winter. The region is characterized by extensive agriculture with small farms (82 % of farms are smaller than 5 ha) and a low human density (30 people/km²; Demographic Yearbook of Poland 2009).

Knyszyn Forest [KF] (53°02′–53°21′N, 22°55′–23°51′E) is situated 40 km north of the Białowieża Forest and covers 1,270 km^2^ (Fig. 1). Tree stands, dominated by pine and spruce, cover 80 % of the Forest. Open areas within the forest (mainly glades and meadows) occupy 11 % of the area (Krasińska and Krasiński 2007). From the north and the east the Forest is surrounded by arable lands (68.5 % of area) with fertile soils and intensive farming, pastures and meadows (12.8 %) and woodlands (16.4 %). The main cultivated crops are rye (21 % of sown areas), oat (21 %) and potatoes (8 %). The proportion of arable lands covered by winter grains and rape is 30 %. This is a typical agricultural region with large and specialised farms (69 % of farms are larger than 20 ha). Human density in the area is 35.5 people/km² (Demographic Yearbook of Poland 2009).

The climate of both Knyszyn and Białowieża Forest is transitional between Atlantic and continental types. Mean annual temperature is 7 °C in BF and 8.5 °C in KF. The warmest month is July (average 18.4 °C in BF and 18 °C in KF), and the coldest is January (average −4.8 °C in BF and −3.5 °C in KF) (Górniak and others 2008; Jędrzejewska and Jędrzejewski 1998). The average vegetation period is 200–210 days. Snow cover lasts from 60–96 days in BF and from 85–90 days in KF. During the study period, max. recorded depth of snow in BF was 55 cm in 2005 (mean monthly max. was 27 cm). Annual precipitation averages 610 mm in BF and 631 mm in KF (Górniak 2000).

### Characteristics of Bison Populations

The population in BF, created in 1952, was the first re-introduced bison population in Europe and is currently the largest. It increased between 2000 and 2010 from 306 to 473 individuals (Raczyński 2000–2010). To reduce the damage to tree stands and farm crops and reduce migration out of the Forest, bison in BF are regularly fed with hay and silage at several winter feeding sites within the forest. However, 15–20 % of bison spend the winter outside of the forest in the neighboring forest agriculture areas (Fig. 1). Compensation for bison damage in this region was first paid in the early 1990’s.

The bison population in Knyszyn Forest was initiated in 1973 by a single migrating male from Białowieża Forest and five other bison, which had been re-introduced there by foresters (Krasińska and Krasiński 2007). The population has increased from 32 bison in 2000 to 98 in 2010 (Raczyński 2000; 2010). Bison herds occupy the northeastern part of the Forest (Krasińska and Krasiński 2007). During the growing period bison utilize only 6 % of the total area of the Knyszyn Forest (Fig. 1). Supplementary feeding is limited and bison only occasionally utilize one winter feeding site created in the Forest. In late autumn, 90–100 % of bison migrates out of the forest and utilizes agricultural areas till spring, only occasionally returning to the forest area (Krasińska and Krasiński 2007). Compensation for damage caused by bison in this area was first paid in 1998.

### Data Collection and Analysis

Crop damage data was collected from damage assessment protocols provided by the Regional Directorate for Environmental Protection (RDEP). The protocols were prepared after notification on damage by farmers. All recorded cases of damage were investigated by experienced RDEP agents to identify the ungulate species causing the damage, the area and the rate of crop damage. Identification of species was based on direct observations, tracks and other signs of activity (resting beds, feces). Tracks of bison are easy identified, as they are much larger than other ungulates, except for moose, which is very rare in the area and do not utilize farmlands. On the basis of the estimated volume of damaged crops and the current market price of the crop, a compensation amount was estimated. Protocols contained data on location and dates of damage occurrence, character of damage (foraging, trampling, bedding, wallowing), types of damaged crops and amount of compensation paid to the aggrieved farmer.

Between 2000 and 2008, damage was recorded on 295 farm properties (from 1 to 30 records on each). In total we analyzed 634 cases of bison damage in the Białowieża Forest region and 449 cases in the Knyszyn Forest area. We excluded from the analysis four cases of domestic animals which were killed by bison (two dogs, one cow and one horse), as the state only pays compensation for damage caused by bison to cultivations, agricultural produce or private forest (Nature Conservation Act 2004). Additionally, we included data on damage number and compensation costs in 2009–2010.

Locations of crop damage were digitized and geo-referenced using maps available from the www.geoportal.gov.pl web site (http://maps.geoportal.gov.pl/webclient) and imported into a Geographic Information System [GIS]. Using a distance estimator in MapInfo Professional (Version 8.0) we calculated the distance from the location of damage to the nearest woodland patch and to the edge of the continuous forested area of the Białowieża Forest and the Knyszyn Forest. These distances were compared to the distances to 100 random points generated for both areas covered by damage locations (excluding points that fell inside the forest areas) using the Excel 2007 spreadsheet location software. Distribution and aggregation of depredation locations was analyzed using the Kernel (fixed) method (Rodgers and Carr 1998; Worton 1989) and the nearest neighbor dispersion analysis (Krebs 1989) using the Biotas 2.05 (Ecological Software Solutions, USA) program. In the nearest neighbor analysis, index of aggregation (*R*) was estimated on the basis of mean observed (*r*_*a*_) and expected (*r*_*e*_) distances to nearest neighbor using the formula: *R* *=* *r*_*a*_ */r*_*e*_. In a regular distribution, *R* is significantly greater than 1, whereas in an aggregated distribution *R* is significantly less than 1. To test for significant deviation from a random we used the *z*-test. The influence of weather conditions (snow cover depth, ambient temperatures) on the frequency of bison damage was only analyzed for winter periods (December–March) in Białowieża Forest. This was because the detailed weather data on the mean monthly temperatures and snow cover depth were only available for this area. Data was obtained from the weather station in Białowieża.

We calculated the bison’s selection to different crops using Jacobs’ electivity index, *D* (Jacobs 1974): *D* *=* (*r* *−* *p*)/(*r* *+* *p* *−* *2pr*), where: *r* is the number of the given type of damaged crop records as a fraction of the total number of damage records; *p* is the fraction of the area covered by a given crop in the total sown area (Statistical Yearbook of Agriculture 2009). *D* ranges from −1 (the strongest negative selection) to +1 (the strongest positive selection), with 0 being random utilisation.

## Results

**Fig. 2:**
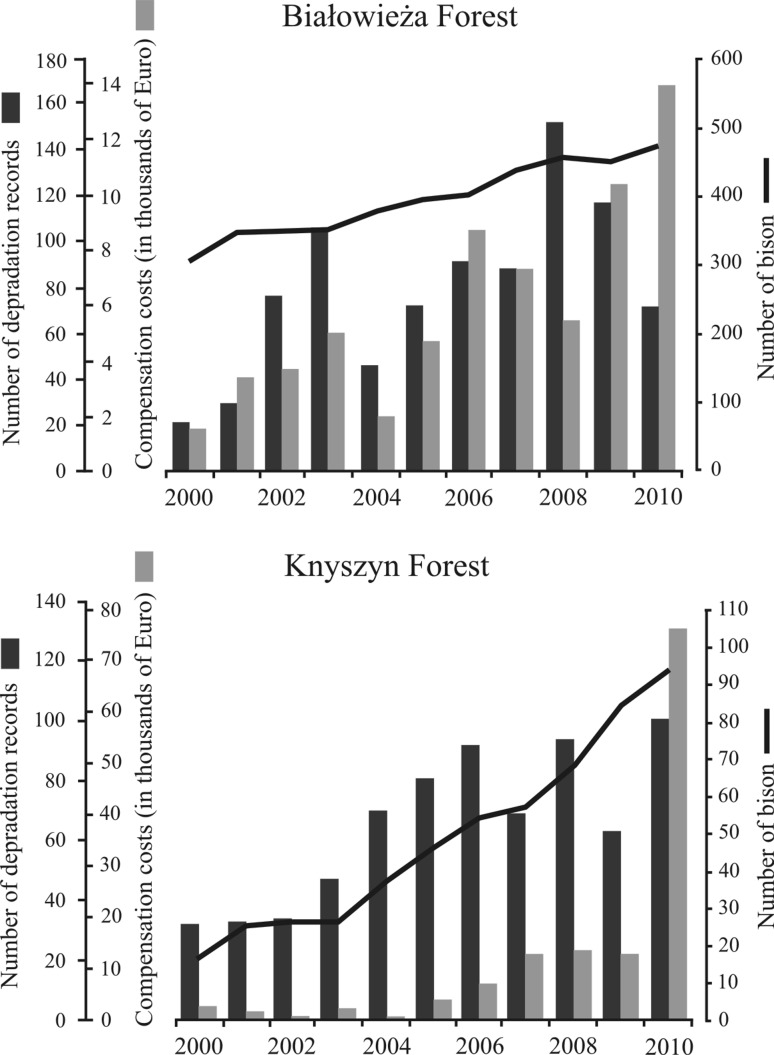
Amount of compensation, number of depredation records and number of bison in Białowieża Forest and Knyszyn Forest in 2000–2010

The overall cost of bison damage for the period 2000–2010 was 784,634 PLN (€ 196,200), 71,330 PLN (€ 17,800) per year on average. The largest amount of compensation for both regions was registered in 2009 and 2010, in total 92,140 PLN (€ 23,000) and 361,690 PLN (€ 90,400) respectively (Fig. 2). Despite the large difference in bison numbers in both Forests, between 2000 and 2010 the mean annual number of instances of damage was comparable: 79 ± 38 (mean ± SD) in Białowieża and 65 ± 26 in Knyszyn region, while mean annual compensation costs was twofold higher in KF (€ 12,000) than in BF (€ 6,000). The amount of damage increased during the study period (11 years) in both forests and was positively correlated with bison number (*r* ^2 ^= 0.41; *n* = 11; *P* = 0.033 in BF; *r* ^2 ^= 0.61; *n* = 11; *P* = 0.004 in KF) (Fig. 2).

**Fig. 3:**
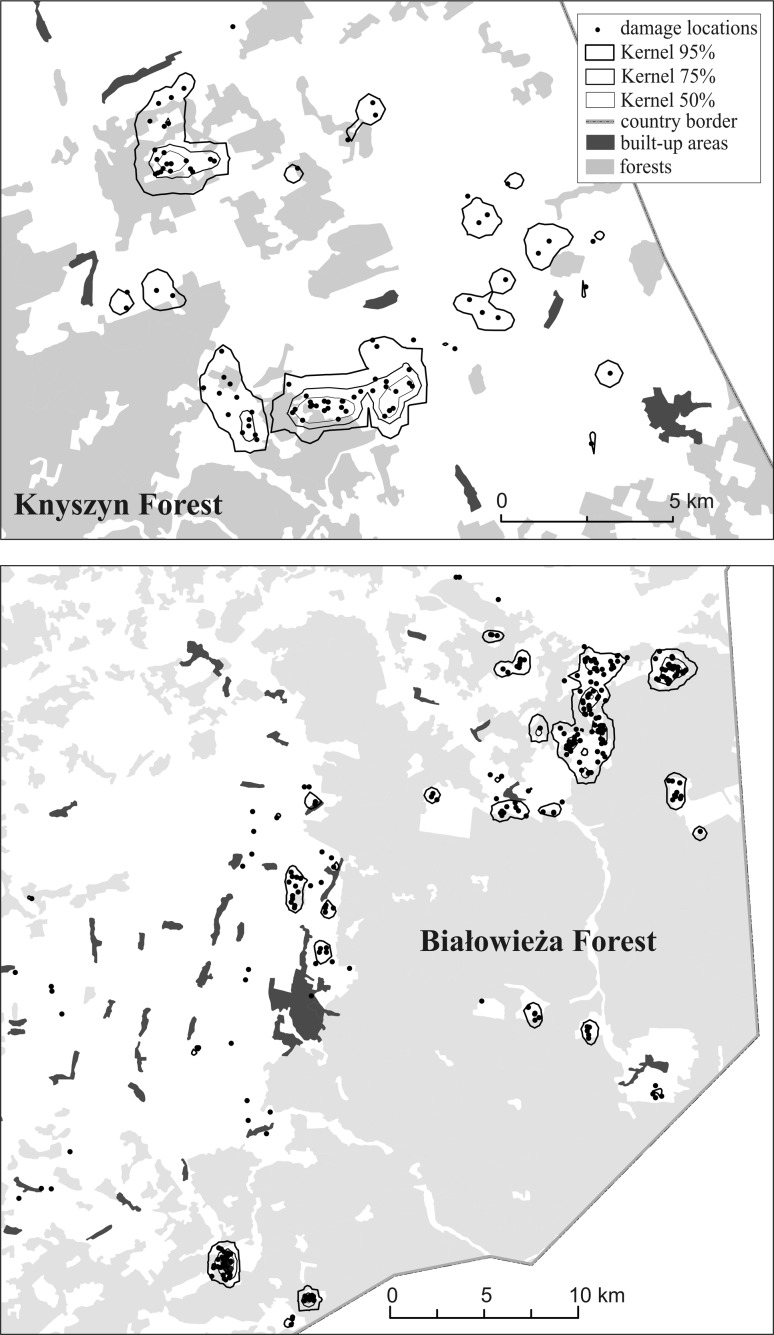
Distribution and core areas of European bison damage in the vicinity of Białowieża Forest and Knyszyn Forest

Depredation locations were distributed in the area of 996.1 km^2^ (MCP 100 %) in BF, and 141.9 km^2^ in KF and 73.6 km^2^ and 29.1 km^2^ respectively, when the Kernel (95 %) method was used (Fig. 3). Core areas of damage distribution estimated with Kernel 50 %, covered 3.5 and 1.1 km^2^, i.e. 4.8 and 3.8 % of Kernel 95 % ranges. The mean distances to the nearest neighbor were 410 ± 26 m in BF and 500 ± 30 m in KF and were lower than expected (984 and 656 m, respectively). Index of aggregation (*R*) was 0.42 and 0.76 respectively, which indicates a tendency towards aggregation. Pattern of damage distribution for both areas significantly differed from random (*z* = −21.7 and −5.25 respectively).

The distance between the locations of damaged crops and the edge of the continuous forest varied from 0 to 14.9 km (average 1.2 ± 2.0 km) in BF and from 0 to 8.1 km (average 2.1 ± 2.3 km) in KF. Most damage (45.2% in BF and 36.6% in KF) occurred close (0–0.5 km) to the continuous forest edge but in KF it also ranged from 1.1 to 5.0 km (37.3 %) (Table 1). As a result, distribution of damage in relation to the edge of main forest complex significantly differed between both Forest areas (*χ* ^2 ^= 18.9, *P* = 0.0003). When calculated in relation to the nearest forest patch, most of the damage (80,4 % in BF and 68,7 % in KF) was localized to the area up to 0.5 km away from the forest patch and were different for both regions (*χ* ^2 ^= 14.9, *P* = 0.0006). A decreasing amount of damage was recorded with increasing distance from the forest patch (Table 1). When the distribution of damage locations was compared with randomly selected locations we found different patterns in both Forests. In BF, distances from damage locations and random points to the edge of continuous forest did not differ significantly but were significantly different when compared to distances to the nearest forest patch (Table 1). In the Knyszyn Forest, differences were significant for distances of damage to both, the forest edge and the nearest forest patch (Table 1).

**Table 1:** Comparing the distribution of crop damage by European bison and random points in the vicinities of Białowiea Forest and Knyszyn Forest

	Percentage of crop damage locations																
	Białowieża Forest									Knyszyn Forest							
Distance (km)	To the nearest forest patch				To the main forest complex					To the nearest forest patch				To the main forest complex			
	recorded		random		recorded			random		recorded		random		recorded		random	
<0.5	80.4		53.9		45.2			38.2		68.7		53.5		36.6		18.2	
0.5–1.0	14.8		19.6		22.8			17.6		11.1		17.2		12.7		5.1	
1.1–5.0	4.8		24.5		28.0			41.2		20.2		29.3		37.3		43.4	
5.1>	–		2.0		4.0			2.9		–		–		13.4		33.3	
Statistics	χ^2 ^= 32.05; *p* < 0.0001				χ^2 ^= 7.47; *p* = 0.0585					χ^2 ^= 9.31; *p* = 0.0095				χ^2 ^= 42.68; *p* < 0.0001			
Mean ± SD	0.4 ± 0.6		0.9 ± 1.2		1.2 ± 2.0			1.5 ± 1.6		0.5 ± 0.6		0.7 ± 0.6		2.1 ± 2.3		3.7 ± 3.2	

**Fig. 4:**
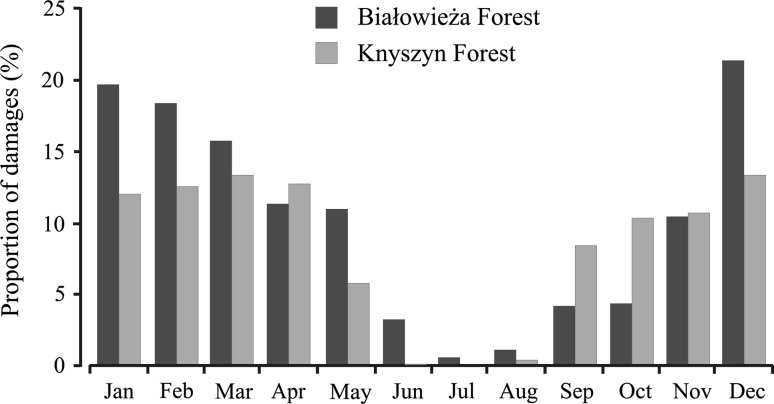
Seasonal pattern of crop depredation by European bison in the vicinity of Białowieża Forest and Knyszyn Forest

Bison depredation had seasonal character. Most of the damage (57.1 % of cases) was reported in winter (December–March) and only 2.5 % of all damage was registered in summer (June–August) (Fig. 4). The monthly distribution of damage cases differed significantly between the two areas (*χ* ^2^ = 53.901, *P* < 0.0001). We have not found a significant influence of mean monthly snow depth and temperature during winter (December–March) on the amount of damage recorded in BF (*r* ^2^ = 0.029 and 0.073 respectively, *n* = 36 months; *P* > 0.05).

**Fig. 5:**
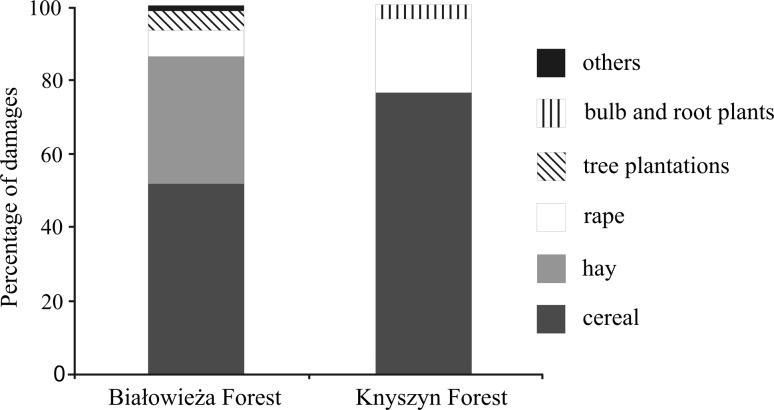
Differences in the structure of farm crops depredated by European bison in area of Białowieża Forest and Knyszyn Forest

In total 19 types of cultivation were damaged by bison. The crops damaged most frequently were cereals (61.3 % of damage cases), followed by rape (12.7 %) and hay (20.1 %) (Table 2). Among cereals rye (65.4 % of damaged grains) and triticale (21.4 %) were damaged most frequently. We recorded significant differences in the proportion of different crops damaged by bison in both areas (χ^2^ = 1189.3, *P* < 0.0001) (Fig. 5). In the Białowieża Forest, the main crops damaged were cereals (51.3 %) and hay (34.1 %). The proportion of cereals in Knyszyn region was higher (75.6 % of damage cases) and the second most frequently depredated crop was rape (19.3 %) (Fig. 5). The average size of damaged agriculture field was 1.6 ± 7.1 ha (range: 0.01–10.1 ha) in Białowieża and 25.7 ± 48.1 ha (range: 0.1–135.5 ha) in Knyszyn region.

**Table 2:** Proportion of different farm crops damaged by European bison in the vicinity of Białowieża Forest and Knyszyn Forest

Type of damaged crop	Number of damage records	%
Cereal	706	61.3
Rye	461	40.0
Triticale	151	13.1
Wheat	44	3.8
Oat	38	3.3
Barley	12	1.1
Hay and silage	231	20.1
Rape	146	12.7
Tree plantations	30	2.6
Bulb and root plants	11	0.9
Other crops (strawberries, maize, vetch, lupine and buckwheat)	28	2.4
Total	1152	100.0

**Fig. 6:**
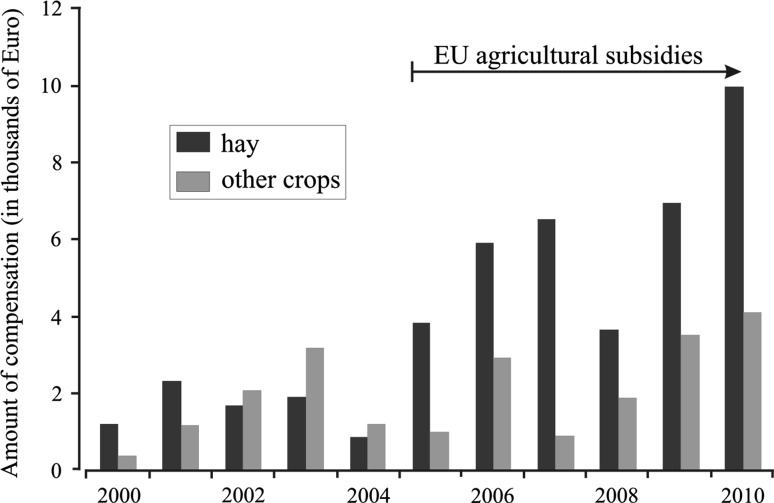
Influence of the EU agricultural subsidies on compensation costs for crops damaged by bison in the Białowieża Forest area in 2000–2010

Detailed analysis of the annual distribution of damage to hay and other crops in BF showed that the average compensation for hay damaged by bison between 2000 and 2004 was significantly lower than in following years (*z* = −2.6; *P* = 0.008) (when EU subsidies became available in Poland), while the amount of compensation paid for other crops stayed on the same level (*z* = −0.6; *P* = 0.52) (Fig. 6).

Comparison of the proportion of different crops damaged by bison with the availability of crops based on their proportion in the total sowed area, revealed a very strong selection of rape (Jacobs’ index, *D* = 0.89 and *D* = 0.99, respectively) and rye (*D* = 0.58 and *D* = 0.50, respectively) in both, Białowieża Forest and Knyszyn Forest. Bison avoided crops like barley and oat (Table 3).

**Table 3:** Selection of farm crops by European bison in northeastern Poland. Jacobs’ index *D* varies from −1—strong avoidance to 0— random selection to +1—strong preference

	Selectivity index *D*	
Cultivation	Białowieża Forest	Knyszyn Forest
Rape	0.89	0.99
Rye	0.58	0.50
Triticale	−0.13	0.48
Wheat	−0.86	0.41
Oat	−0.33	−0.83
Barley	−1.0	−0.33

## Discussion

We found that European bison widely used agricultural areas, despite the fact that the species has been recognized as a forest specialist (Sokolov 1959; Sztolcman 1926). As showed by Kerley and others (2012), bison populations have a natural tendency to disperse into open habitats. Nearly 70 % of the bison populations that were initially introduced into forest habitats expanded their distribution range to neighboring open areas, often dominated by farmlands (Kerley and others 2012). The origins of bison, its morphological adaptations, diet and recent data on habitat use suggest that bison are large grazers adapted to mixed or opened habitats (Balčiauskas 1999; Gębczyńska and others 1991; Kerley and others 2012; Kowalczyk 2010; Mendoza and Palmqvist 2008).

Bison depredation of farm crops is still a local issue previously only reported from Lithuania (Balčiauskas 1999). However, due to an increase in the total world population (6 % annually), the pace of new re-introductions, range expansion of re-introduced populations and potential creation of populations (e.g., in Romania, Hungary, Germany, Denmark), this problem will most likely increase and human-bison conflict is expected to emerge in new locations and grow in existing herds. In northeastern Poland, compensation paid for bison damage in 2010 constituted only 8 % of costs induced by Eurasian beavers (*Castor fiber*) (data of Regional Directorate for Environmental Protection in Białystok). However, the amount of compensation paid for bison was increased over 20-fold during the last 10 years. Despite a substantial difference in the number of bison in Białowieża Forest and Knyszyn Forest (473 vs 98 individuals in 2010) a comparable level of damage was recorded. This was due to a comparable number of bison utilizing agriculture areas in both populations. In Knyszyn Forest 90–100 % of all bison penetrate agricultural areas during winter, whereas in Białowieża Forest it is only 15–20 % of the population (Krasińska and Krasiński 2007).

Our analysis showed a relationship between the level of damage and growing bison numbers in both populations. The increasing rate of damage is probably not only a function of the growing bison number but also the extension of distribution ranges, especially in Białowieża Forest. Furthermore, in this area, the re-use of meadows previously abandoned by farmers, increased after accession of Poland to the European Union in 2004. This was an effect of the EU subsidies which became available to Polish farmers for maintaining meadows and arable lands. Hay collected by farmers from subsidized meadows was stored at site and thus became available to bison roaming on the edges of the Forest. The volume of stored hay increased several times in comparison to the years before Polish accession to the EU. In effect, the number of damage and amount of compensation paid for damage to hay increased threefold after 2004, while compensation claims for other crops did not change. Therefore, compensation growth in the Białowieża region after 2004 was caused indirectly by the EU agricultural subsidies. In the Knyszyn region the damage had a different character. Bison damage there incurs serious economic loss to farmers, as this area is characterized by intensive agriculture and farms that are continuously growing in size. In this region, 90 % of compensation costs in 2010 (€ 69,000 of € 76,400) were paid for rape depredation.

Most of the damage was concentrated on relatively small and specific areas which overlapped with the winter ranges of several bison herds. These herds selected winter cultivations of cereals and rape (in the vicinity of Knyszyn Forest and west from Białowieża Forest) or areas of meadows with numerous haystacks (north from Białowieża Forest). Therefore, bison utilization of agricultural areas is related to distribution of attractive food resources.

The prevailing area of damage in agricultural regions was localized in the close vicinity of woodland patches (<0.5 km), which reflects the significance of these as cover for bison. Numerous ungulate species often forage in open habitat but rest under forest cover (Germaine and others 2004; Mysterud and Østbye 1995; Ockenfels and Brooks 1994). Bison usually rest and ruminate in forested areas and select more dense stands with low visibility (Rouys 2003; C. T. Schneider, Mammal Research Institute, unpublished data).

Seasonal patterns of farmland utilization by bison result from availability of vegetation biomass in forest habitats (Krasińska and others 1987; Krasiński and others 1994). During the growing season, forest habitats are sufficient foraging grounds for bison (biomass of vegetation varies from 83 g/m^2^ in coniferous forests to 304 g/m^2^ in wet deciduous forest) (R. Kowalczyk, Mammal Research Institute, unpublished data) and offer good cover for resting, rumination and calving. However, at the beginning of September, the herbaceous vegetation decays and there is a very low biomass in a forest available for large grazers (Jędrzejewska and Jędrzejewski 1998). To cope with the seasonality of food resources, bison migrate to winter feeding sites or to agriculture areas, where winter cultivations or hay stored on meadows may supply their energy requirements.

Different seasonal pattern of crop depredation was observed in both areas. This was probably related to the different productivities of the forest floors in Knyszyn Forest and Białowieża Forest. In the mostly coniferous and drier Knyszyn Forest, vegetation has already decayed in late summer. Bison are therefore forced to migrate to agriculture areas and the level of depredation there is quite stable from September until April. In Białowieża Forest, mowed meadows within and around the forest area and the wet deciduous forest has lush vegetation until November (Falińska 1973), so there is a delayed migration of bison compared to the Knyszyn Forests. Additionally, in late autumn, some bison herds in Białowieża consume hay stored in roofed haystacks at winter feeding sites. Due to the large distances from fodder storage facilities, the fodder in feeding sites on the edges of the forest is supplemented much less frequently than in its centre (Kowalczyk and others 2011), so bison from those areas often migrate to neighboring agriculture areas to search for food.

The foraging decisions of European bison, probably in the same way as its close relative the American bison, maximise their short-term rate of energy intake (Fortin and others 2002, 2003). European bison prefer the winter cultivations of rye and rape which are characterized by high nutritional value in comparison to hay offered is supplementary sites (Jankowska-Huflejt and others 2004; Jankowska-Huflejt and Wróbel 2006) or vegetation available in forests habitats during winter. Additionally, cereals have the highest content of minerals in vegetative mass during its early growth phases (winter), which decrease in consecutive phenological phases (Barczak 2008; Czarnowska 1975), therefore cereals and rape are important sources of nutrients especially in winter. When supplementary fodder is not available, bison may increase browsing (Kowalczyk and others 2011), but most often migrate to agriculture areas, where they stay till spring, as observed in Knyszyn and some parts of Białowieża Forest.

Differences in the structure of crops damaged by bison in both areas were due to differences in the structure of farm crops and agriculture intensity. Areas neighboring to Knyszyn Forest are characterized by more intensive agriculture, with large cultivations of rape and cereals, so those crops that are the main ones damaged by bison in that area. By contrast, areas adjacent to Białowieża Forest are characterized by a higher proportion of pastures and meadows and more extensive agriculture (Statistical Yearbook of Agriculture 2009). Moreover, in the Białowieża region hay is traditionally stored directly on the meadows in roofed or unroofed haystacks, what is not practising in Knyszyn region.

Snow cover can regulate access to food resources during winter as well as affecting energy expenditures and the locomotion rate (Fancy and White 1985; Rominger and Oldemeyer 1990). Winter severity and heavy snow may also reduce herbage consumption by large ungulates (Christianson and Creel 2007). In our analysis weather conditions did not influence crop depredation by bison, what indicates that snow conditions were still below the limiting threshold for bison. However, it was found that during mild winters with shallow snow cover bison increase their ranging behavior (Krasińska and others 2000).

To decrease the level of human-bison conflict in the Białowieża region, it is necessary to create additional opportunities for long-term local profits (higher than that paid as compensation) based on wildlife conservation. An alternative for unprofitable agriculture as a source of revenues here could be an ecotourism, which is listed as the most important advantage of living next to the protected areas (Archabald and Naughton-Treves 2001). Low human density and well preserved forest habitats create an ideal condition for wildlife tourism. In fact, farming activity almost collapsed within and around the Białowieża Forest. Due to iconic status of the species and increasing tourist interest (140,000 visitors annually), the bison became a driver of local development.

Damage compensation programs are a widely implemented method in many countries for mitigating wildlife-human conflicts (De Klemm 1996). The Polish State is also liable for damage caused by protected species such as beavers, lynx, wolves, bears and bison to arable crops, private forests and livestock. However, in other countries where bison are present such as Belarus, Russia and Ukraine, compensation is not offered to farmers, which results in low acceptance of this large herbivore or even decline of the population due to uncontrolled hunting and poaching, as observed in Ukraine (Kerley and others 2012).

## Management Implications

Large herbivores are a conflict species due to their impact on vegetation (including farm crops) and limited space in fragmented and densely populated areas. Although some authors indicated potential conflict between the bison and farming (Kuemmerle and others 2011), this study is the first one addressing the increasing problem of human-bison conflict in re-introduced populations of the species and analyzing long-term data on crop depredation. The problem of crop depredation probably occurs in the majority of growing and expanding bison populations, however, it may be driven by different factors (e.g. habitat structure, management practices, population increase, intensity of agriculture) and is not monitored and is often neglected in post-Soviet countries (such as Belarus, Russia or Ukraine). Analysis of data from northeastern Poland shows that the process is dynamic and difficult to manage. Seasonal migrations of bison to agriculture areas, which constitute attractive foraging grounds for large grazers, results from strongly depleted food resources in forest habitats in winter.

To minimize human-bison conflict, cooperation between managers, farmers and local stakeholders is required to develop management strategies adjusted to local conditions. Apart from the compensation provided by the State, which seems to be obligatory to mitigate human-bison conflicts, the following actions can be proposed:

1. Provision of winter foraging grounds for bison within forests or their vicinity in forage base (such as mowed meadows with hay left as supplementary fodder for bison and green forage base like crop cultivations sown especially for bison on forage plots);
2. Monitoring the population growth and encouraging bison to disperse to areas of low risk of conflict;
3. Protection of valuable crops or areas with high concentration of depredation (fencing, electric fencing);
4. Regular deterrence of bison herds foraging on agriculture crops to increase their vigilance and mobility and decrease damage concentration;
5. Creation of a buffer zone (at least 0.5 km) around forested areas occupied by bison (repurchasing and inclusion of adjacent agriculture areas and transformation into meadows);
6. Steering bison seasonal migrations routes using short diversionary fences (see Fortin and others 2010).

Supplementary feeding used to prevent damages to farm crops and reduce migrations needs to be reduced or modified due to negative effects on the populations, such as increase of parasitic load and drop of bison condition (Hayward and others 2011; Kowalczyk and others 2010; Pyziel and others 2011; Radwan and others 2010).

The re-introduction of the European bison needs to be based on science-based guidelines. The question is whether there is still enough space for this large herbivore in modern Europe with its highly fragmented natural habitats and numerous barriers. Favorably for bison, in some less populated areas of eastern Europe (e.g. Eastern Poland, Belarus, Russia or Ukraine), extended areas of abandoned farmland appeared in vicinity of forested areas (due to political system transformation and economic changes). Such areas (mosaics of woodland and meadows, former military areas) are suitable for the European bison and guarantee a low level of human-bison conflict. The European bison is an iconic species, a relict of ancient times and one of the last large mammals which have survived to present times in Europe. It is also a great tourist attraction and a driver of sustainable development in areas inhabited by these large animals. Human-bison conflict needs further investigation, as in some areas the problem is currently increasing or will increase in the near future. Thus, management strategies should be aimed at mitigating human-bison conflicts by way of science-based adaptive management.
